# The rise of a digital immune system

**DOI:** 10.1186/2047-217X-1-4

**Published:** 2012-07-12

**Authors:** Michael C Schatz, Adam M Phillippy

**Affiliations:** 1 Simons Center for Quantitative Biology, Cold Spring Harbor Laboratory, Cold Spring Harbor, NY, 11724, USA; 2 National Biodefense Analysis and Countermeasures Center, Frederick, MD, 21702, USA; 3 Center for Bioinformatics and Computational Biology, University of Maryland, College Park, MD, 20742, USA

## Abstract

Driven by million-fold improvements in biotechnology, biology is increasingly shifting towards high-resolution, quantitative approaches to study the molecular dynamics of entire populations. One exciting application enabled by this new era of biology is the “digital immune system”. It would work in much the same way as an adaptive, biological immune system: by observing the microbial landscape, detecting potential threats, and neutralizing them before they spread beyond control. With the potential to have an enormous impact on public health, it is time to integrate the necessary biotechnology, computational, and organizational systems to seed the development of a global, sequencing-based pathogen surveillance system.

## The “mega-genomics” era

If the last two decades marked the beginning of the genomics era, starting with the sequencing and publication of the first free-living organism in 1995 and then the human genome in 2001, the current decade marks the beginning of the “mega-genomics” era, where large numbers of genomes are analyzed with diverse, sequencing-based assays to infer molecular diversity and dynamics of life. Examples include projects to determine the molecular basis of complex human diseases such as cancer [1], to study the incredible diversity and function of the human microbiome [2], to rapidly identify the origins of pathogen outbreaks [3], and to generally develop a deeper understanding of the living world through the increasing use of large-scale sequencing.

These breakthroughs are driven by a shift from single-reference genomics to more quantitative, population-wide analyses. Biology has moved beyond developing a merely qualitative understanding of cellular and evolutionary processes, and now strives for base-pair resolution and predictive models of biological systems and disease. This has been enabled through the combination of dramatically improved biotechnology, computer technology, algorithms, and statistical models. Through sophisticated protocols and assays, sequencing is no longer limited to just reading DNA, but has been creatively adapted to measure transcript abundance, protein-DNA binding patterns, and the three-dimensional configuration of DNA or RNA, among others (see [4] for a overview of available applications). Sequencing throughput and costs have improved by more than a million-fold, and these advances have risen alongside similarly radical advances in computational technology and algorithm sophistication [5].

Amazingly, there seems to be no end to the exponential capability growth we have witnessed, and vendor roadmaps continue to project breakneck innovation well into the next decade. Worldwide sequencing capacities currently exceed 15 petabases per year, and compute clouds with seemingly infinite capacity can now be rented on demand. On the sequencing side, real-time, single-molecule sequencing has been achieved by Pacific Biosciences, and Oxford Nanopore has promised to deliver a mobile, disposable sequencing device the size of a thumb-drive [6]. With equally amazing advancements happening every year, it is virtually certain that the confluence of cheap sequencing and “big data” computer science will enable many new, digital forms of biology.

## A digital immune system

One exciting application of digital biology with the potential to have enormous public health impact is the “digital immune system.” The term, coined by David Lipman of NCBI, draws an analogy between computing and biology—a recurring technique of computational scientists (viruses, genetic algorithms, neural networks). A digital immune system would work in much the same way as an adaptive, biological immune system: by observing the microbial landscape, detecting potential threats, and neutralizing them before they cause widespread harm. This simple strategy, effectively tested over millions of years, can now start to be replicated *in silico* with the combination of distributed “sensor” sequencing and bioinformatics—where a network of mobile sequencing devices serves a real-time stream of microbial genomes to a global compute cloud for analysis.

An effective immune response relies on the ability to differentiate normal from abnormal. In the digital realm, this ability will rely on extensive knowledge of microbial diversity. However, unlike the macroscopic world where outliers can often be easily recognized, microbial diversity is less well characterized, with only a small fraction of the world’s microbes ever sequenced [7]. It is difficult to characterize an emerging outbreak, for example, when only a handful of known genomes exist. Effective pathogen detection and response requires a complete catalog of genomic diversity, antibiotic resistance, and virulence across both temporal and geospatial dimensions. This must be achieved by sequencing and archiving huge numbers of microbial genomes, both from clinical cases and known environmental reservoirs, on a continual basis.

Just as an immunological memory improves with each exposure, genome databases will also expand and improve over time as new outbreaks and environments are analyzed, but only if this digital memory is properly managed. Standardized sequences and metadata must be made freely available in real-time and on a global scale, requiring a daunting level of cooperation. The primary nucleotide archives NCBI, EMBL and DDBJ are obvious candidates for this task, but these archives must rapidly adapt to the new era of population sequencing. The current database models are outdated; the number of genomes being submitted lags far behind the genomes being sequenced and those submitted often lack essential metadata. Barriers must be eliminated and new incentives structured to encourage the submission of usable, large-scale data: “more data, faster” should be the guiding principle and the minimum metadata of “what, where, when” (sequence, location, time) must be reliably captured.

An explosion of openly available microbial genomes, linked with temporal and geospatial metadata, would undoubtedly lead to new discoveries in epidemiology and ultimately lead to more predictive biology. Open data sharing has already reduced outbreak attribution to a matter of weeks, as evidenced by the “crowd-sourced” responses to the swine flu [8] and *Escherichia coli* O104:H4 [9] outbreaks, while the NIAID Influenza Genome Sequencing Project, spearheaded by The Institute for Genomic Research (now JCVI) [10], has hinted at the predictive potential. Influenza research has exploded with the ongoing generation and release of these genomes, spawning many follow-on studies and predictive models that have shown, among other things, that seasonal influenza severity can be predicted by the genetic diversity of the circulating strains [11]. The potential power of expanding such surveillance efforts is extremely compelling and would drastically shape the future of infectious disease—potentially stopping the next outbreak before it happens.

The technology necessary to implement these proposals is imminent, although a few significant obstacles remain. Computational hurdles, while daunting, are perhaps the most manageable, with computer science, high-energy physics, and astronomy already leading the big-data charge. Rather, a more pressing need is for inexpensive and portable sequencing devices that can act as the sensors in a distributed, real-time sequencing network—just as atmospheric sensors feed real-time data to sophisticated weather modeling programs. These sequencing sensors would also require significant advances in sample preparation to allow easy nucleic acid extraction direct from any sample, so that they could be widely deployed and operated by health care providers rather than specialists.

As the necessary technologies continue to grow to address these needs, there is no need to wait. Recent studies have shown that implementing this vision on a limited scale can yield tremendous insight (e.g. [12]). In addition, many universities and hospitals already perform routine pathogen sequencing and environmental screens. What is needed now is an organized effort towards making these genomes as widely available as possible to enable a digital immune system with the potential to drastically advance human health.

## Competing interests

The authors declare that they have no competing interests.

## Authors’ contributions

Both authors contributed equally to the drafting of the manuscript. Both authors read and approved the final manuscript.
